# Supplementary figures and images for: EFFECTS OF TOLL-LIKE RECEPTOR 3 – DEPENDENT IMMUNE ACTIVATION IN MICE ARE SEX- AND TISSUE- SPECIFIC: IMPLICATIONS FOR ALCOHOL USE DISORDER
Source: bioRxiv. 2026 Apr 24:2026.04.21.719957. Preprint. [Version 1] doi: 10.64898/2026.04.21.719957 (PMC13131485; doi:10.64898/2026.04.21.719957)

Supplementary Figure 1.

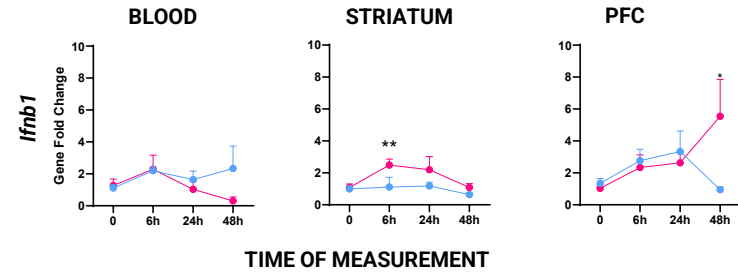

Supplementary Figure 2.

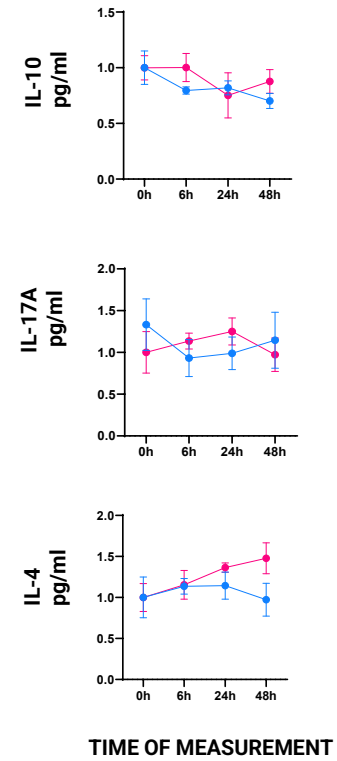

Supplement: Supplement 1 — Supplementary Figure 1: Sex-specific expression of blood, striatum, and PFC Ifnb1 0, 6, 24, and 48 hours after PIC injection in FVB/B6 F1 hybrid mice. Supplementary Figure 2: Sex-specific expression of immune proteins in striatum at 0, 6, 24, and 48 hours after PIC injection in FVB/B6 F1 hybrid mice. [file media-1.pdf]
